# Mental Health Benefits of Breastfeeding: A Literature Review

**DOI:** 10.7759/cureus.29199

**Published:** 2022-09-15

**Authors:** Zachary Tucker, Chasity O’Malley

**Affiliations:** 1 Biomedical Sciences, Nova Southeastern University, Davie, USA; 2 Medical Education, Nova Southeastern University Dr. Kiran C. Patel College of Allopathic Medicine, Fort Lauderdale, USA; 3 Medical Education, Wright State University Boonshoft School of Medicine, Dayton, USA

**Keywords:** postpartum mothers, breastfeeding duration, postpartum mental health, postpartum depression, mental health, breastfeeding

## Abstract

Pregnancy is typically viewed as a time of emotional well-being for prospective mothers, but for some, this period can negatively impact mental health. However, the relationship between postpartum mental health and breastfeeding is not clearly understood. Considering that many health authorities recommend breastfeeding, clearly defining this relationship is important. This review aims to illustrate the effects that breastfeeding has on the mental health of postpartum mothers. An extensive computerized search was performed through databases of PubMed, CINAHL, and Medline. All studies conducted to determine the effects of breastfeeding on mental health were screened and included in this review. Search terms related to breastfeeding, postpartum, and mental health were used. This review on breastfeeding and postpartum depression (PPD) begins by discussing the correlation between lactation and the maternal stress response. Another component discussed is the duration of breastfeeding and its importance in limiting PPD symptoms. The review then shifts to focus more on the psychological aspects of breastfeeding, notably on changes to the sleep-wake cycle and mother-infant interactions. The final part of the review emphasizes the danger that early breastfeeding cessation imposes on a mother’s mental health, portraying how prenatal and early-onset postpartum depression may lead to early breastfeeding cessation. This composite collection of studies clarifies the importance of breastfeeding in reducing the incidence and severity of maternal postpartum depression.

## Introduction and background

It has been established that breastfeeding provides numerous benefits to the infant and mother, both in the short term and long term. Studies have shown that breastfeeding can protect against various chronic diseases. Mothers who breastfeed tend to have lower rates of obesity, which in turn reduces the risk of developing hypertension, cardiovascular disease, hyperlipidemia, and even certain types of cancer [1]. Breastfeeding also provides a psychoneuroimmunological benefit to mothers that reduces anxiety, which is likely associated with the hormone prolactin [2]. Other studies have discovered that breastfeeding directly decreases the symptoms of depression and that early cessation of breastfeeding eliminates this benefit [3].

The World Health Organization (WHO) and many other authorities recommend that mothers begin breastfeeding within the first hour of life and exclusively breastfeed for six months. The WHO also suggests that mothers continue breastfeeding through age two or beyond [4]. While breastfeeding offers numerous health benefits and is recommended by major authorities, breastfeeding rates in most countries remain low, with only 55.8% of infants still breastfeeding at six months in the United States [5].

There are many possible explanations for this stark difference in mothers who breastfeed in comparison to WHO recommendations. One possible factor that has been explored for this situation relates to a woman’s mental health before and after birth. The perinatal period (from pregnancy to the first year postpartum) has been linked to the development of mental health disorders in women, such as anxiety and mood disorders [3]. A common societal notion that “breast is best” makes some women feel inadequate if they choose not to breastfeed, contributing to the potential development of mental illness in mothers [6]. Prior epidemiological data have shown that at least 20% of women are affected by perinatal depression during pregnancy and the first three months postpartum [7]. The objective of this review is to clarify the effects that breastfeeding has on maternal psychological health.

## Review

Lactation and the stress response

There is a strong correlation between lactation and reduced stress responses, specifically that of cortisol [8]. A study conducted among 10 lactating and 10 non-lactating women discovered that plasma ACTH (p < 0.001), cortisol (p < 0.05), and glucose (p < 0.001) responses to exercise were significantly reduced in lactating women [8]. Furthermore, basal norepinephrine levels were also reduced in lactating women (p < 0.05), indicating that the hormones involved in the stress response are diminished in breastfeeding mothers [8]. Numerous other studies evaluating breastfeeding sessions have confirmed that breastfeeding is associated with a significant decrease in adrenocorticotropic hormone (ACTH) [8-10]. ACTH and the glucocorticoid cortisol play a significant role in an individual’s mental health, notably by controlling anxiety and depression. These two hormones are associated with the activation of the hypothalamus-pituitary-adrenal (HPA) axis, a stress pathway in the brain [11]. Various types of psychological stressors can activate the HPA axis and induce the stress response. The HPA axis increases cortisol secretion to the adrenal cortex, which ultimately leads to increased anxiety [12].

Research has shown that depression may be caused by an increase in the levels of glucocorticoid hormones which activate the HPA axis and lead to symptoms of depression. Activation of this axis may further lead to decreased sleep and self-efficacy [13]. Breastfeeding has been shown to limit the activation of the HPA axis and secretion of glucocorticoids, ultimately reducing symptoms of postpartum depression [2,8,9]. The duration of skin-to-skin contact was also shown to affect maternal cortisol levels; longer durations of skin-to-skin contact led to reduced levels of maternal cortisol, limiting the symptoms of postpartum depression and anxiety [10].

Improved self-efficacy and bonding

Research also shows that breastfeeding improves psychological processes that protect mothers from postpartum depression. Maternal self-efficacy is improved in mothers who breastfeed [14], and self-efficacy is inversely associated with postpartum depression [15], serving an important role in the protection of maternal psychological health. Mothers who breastfed were also found to have higher confidence levels and rated their infants as less irritable during feedings. [14]. Overall, mothers with higher levels of breastfeeding self-efficacy presented lower levels of postpartum depression symptoms [16].

Feeding patterns have been shown to affect mother-infant bonding, with breastfeeding mothers displaying a more established attachment with the infant than non-breastfeeding mothers [17]. In fact, multiple studies have shown that this mother-infant interaction is significantly weakened when the mother is depressed or not breastfeeding [17,18]. Breastfeeding has proven to be crucial in the development of mother-infant bonding, with infants showing significantly more physical contact and vocalizations toward the mother [19,20]. These positive infant reactions help reduce depression in the mother. Researchers have found that the infant’s touch of the mother’s nipple positively influences the mother-infant relationship during the first four days after birth and alters maternal neuroendocrine function [21].

Breastfeeding and sleep

**Figure 1:**
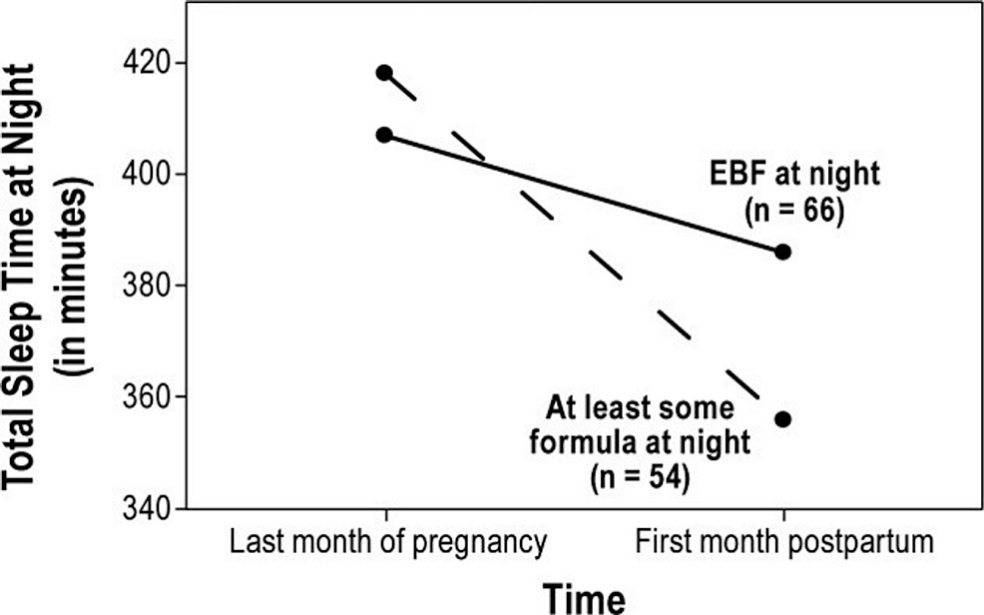
Total sleep time at night over time and by feeding group Field et al. [14]. EBF: exclusive breastfeeding.

Breastfeeding is associated with changes in the sleep and wake cycles for both the mother and the infant. These changes help to reduce fatigue in the mother and may even prevent symptoms of depression. A study investigated the sleeping patterns of postpartum women immediately following delivery and found that breastfeeding women slept on average 2.6 hours longer than women who bottle-fed [22]. In a longitudinal study among first-time mothers, nocturnal sleep at one month postpartum was found to be significantly greater for mothers who exclusively breastfed compared to mothers who used formula at night. This study discovered that mothers who used formula at night suffered nearly three times the amount of sleep loss compared to exclusively breastfeeding mothers [23]. Figure 1 displays the difference in total sleep time at night between exclusive breastfeeding mothers and bottle-feeding mothers, from the last month of pregnancy to the first month postpartum [23]. Figure 1 illustrates a sharp decline in total sleep time for mothers who chose not to exclusively breastfeed [23].

Less sleep at night can negatively affect the mother’s physical and emotional health, increasing the risk of developing postpartum depression [23]. A comparison study found that sleep is also essential for the mother’s milk supply, as there is a potential association between deep sleep and prolactin levels [24]. The study discovered that breastfeeding women had significantly longer phases of slow-wave sleep [24]. The belief is that this occurs due to increased circulating prolactin in breastfeeding mothers, supporting the theory idea that sleep is crucial for proper breastfeeding [24]. A separate study found similar results confirming this theory. The study found that better sleep efficiency was less likely to delay the onset of lactogenesis, further confirming the concept that proper sleep is necessary for breastfeeding [25]. This relationship between sleep and circulating prolactin further emphasizes the importance of sleep in not only maintaining the mother’s mental health but also ensuring adequate breastfeeding ability.

Early cessation of breastfeeding

An important factor in deterring postpartum depression is the initiation and duration of breastfeeding. Researchers have found that prenatal depression is associated with late initiation of breastfeeding, and postnatal depression is associated with a shorter duration of breastfeeding [26]. A study in Taiwan discovered that early breastfeeding is associated with a decreased risk of developing postpartum depression [27]. A smaller study conducted in 2014 showed a significant decrease in postpartum depression scores in women who maintained exclusive breastfeeding for more than three months after childbirth [3]. This same study also reported that women who disliked breastfeeding had a higher risk of developing postpartum depression at two months [3].

The relationship between breastfeeding and postpartum depression is bidirectional; while breastfeeding appears to reduce the incidence of postpartum depression, researchers have also discovered that depression may cause early cessation of breastfeeding. A prospective cohort study analyzed 83 women and found a strong association between postpartum depression (PPD) and early breastfeeding cessation [28]. A separate longitudinal cohort study found that prepartum levels of anxiety and depression were strongly correlated to breastfeeding cessation and that early breastfeeding cessation was predictive of an increase in PPD [29]. Further studies have examined the median duration of breastfeeding for women with and without postpartum depression. A study found that the median duration of breastfeeding was 26 weeks for women with early-onset depression and 39 weeks for women without depression. This stark contrast shows a strong association between the presence of depression and early cessation of breastfeeding [30]. In certain instances, depressed mothers will still attempt to breastfeed. However, depressed mothers who try to breastfeed consistently report experiencing issues and feeling unsatisfied with the process, further strengthening the symptoms of PPD [31].

While psychological factors such as stress, depression, and anxiety can all negatively affect the ability to breastfeed, biology and environmental factors also play a huge role. Numerous health conditions can affect a mother’s ability to breastfeed. Thyroid issues, diabetes, and polycystic ovarian syndrome can all impact hormone levels, disrupting the balance needed to breastfeed [32,33]. Mastectomies and other breast surgeries may destroy the anatomy of the mammary gland, inhibiting a mother’s ability to lactate. [33]. Obesity and diabetes also play a significant role in the inhibition of breastfeeding due to increased progesterone, a hormone stored in adipose tissue that inhibits lactogenesis [33,34]. Women with diabetes are more likely to experience delayed lactogenesis due to lower prolactin concentration [34]. Anatomically, obese women tend to have more adipose tissue in between the ducts of the breast, potentially inhibiting the proper flow of milk [35].

In addition to these health conditions, many women may simply choose not to breastfeed. A study conducted in Hong Kong used a questionnaire among 250 women to assess the reason why first-time mothers chose not to breastfeed [36]. The results showed a variety of personal, cultural, social, and environmental factors that influenced the decision to breastfeed [36]. Table 1 elaborates on the numerous personal factors influencing the decision to breastfeed. Among the personal factors, an overwhelming majority of the women agreed on the importance of the mother-infant relationship as a determining factor for breastfeeding [36]. One hundred seventy-five women also agreed that breastfeeding makes them feel important, emphasizing the role that breastfeeding plays in increasing self-efficacy [36]. Two significant factors that influenced feeding were the mother’s knowledge and attitude toward breastfeeding and the presence or absence of the husband’s support [36].

**Table 1:** Major personal factors influencing breastfeeding among first-time mothers Kong and Lee [36].

Factors	Agree, n (%)	Neither, n (%)	Disagree, n (%)
1. I would feel embarrassed if someone saw me breastfeeding	161 (70)	15 (6.5)	54 (23.5)
2. Breastfeeding is inconvenient	127 (55.2)	21 (9.1)	82 (35.7)
3. Breastfeeding makes me feel run down	112 (48.7)	47 (20.4)	71 (30.9)
4. If I knew more about breastfeeding, I would breastfeed	154 (67)	22 (9.6)	54 (23.4)
5. I am not producing good quality milk	28 (12.2)	69 (30)	133 (57.8)
6. Breastfeeding is economical	199 (86.6)	6 (2.6)	25 (10.9)
7. Breastfeeding is enjoyable	62 (26.9)	75 (32.6)	93 (40.5)
8. Breastfeeding makes the baby closer to me	221 (96.1)	4 (1.7)	5 (2.1)
9. Breastfeeding makes me feel important	175 (76)	26 (11.3)	29 (12.6)
10. Insufficient breast milk is a barrier to breastfeeding	156 (67.9)	32 (13.9)	42 (18.2)
11. I do not think I know enough about breastfeeding	154 (66.9)	36 (15.7)	40 (17.4)
12. Breastfeeding is difficult	83 (36)	46 (20)	101 (43.9)
13. Breastfeeding makes my breasts sag	74 (32.1)	80 (34.8)	76 (33.1)
14. The physical pain and discomfort associated with breastfeeding have discouraged my decision to breastfeed	81 (35.2)	34 (14.8)	115 (50)

A self-reporting study conducted in Arkansas found that many women choose not to breastfeed because of the lack of support [37]. This may include the hospital not teaching the mother how to breastfeed or reasons related to household support [38,39]. Table 1 cites that 67% of the polled women did not breastfeed because they were not educated enough on the matter [36]. Numerous studies call for more individualized support within the hospital, citing a lack of proper procedures and policies as the primary reason for lower breastfeeding rates [36-39].

## Conclusions

The comprehensive literature review summarizes the relevant studies analyzing the effects of breastfeeding on maternal mental health. Studies consistently show that breastfeeding provides countless benefits for both the mother and the infant. Exclusive breastfeeding increases the mother’s self-efficacy and provides protection from symptoms of postpartum depression. Breastfeeding may protect the mother and infant from numerous chronic diseases. Furthermore, breastfeeding reinforces the mother-infant relationship and reduces fatigue in the mother by promoting a proper sleep-wake cycle. These benefits emphasize the importance of breastfeeding in maintaining the mental health of the mother. The primary learning point from this review is the positive effects that successful breastfeeding imposes on the psychological health of the mother. Secondly, screening for early-onset depression will allow mothers to seek support early on and potentially avoid premature cessation of breastfeeding. Lastly, the support systems in place need to be improved. Many studies included in this review highlighted the lack of individualized care, instruction, and support for mothers attempting to breastfeed. Addressing these concerns should theoretically increase breastfeeding rates among mothers and thus limit the incidence of postpartum depression.
